# Supplementary material for: A set of multi-entry identification keys to African frugivorous flies (Diptera, Tephritidae)
Source: Zookeys. 2014 Jul 24;(428):97–108. doi: 10.3897/zookeys.428.7366 (PMC4143993; doi:10.3897/zookeys.428.7366)
Supplement: Supplementary material 4 — Key to Capparimyia [file zookeys-428-097-s004.zip › SF4_ZooKeys_key to Capparimyia/lucid_player/help/help/multimedia_in_the_lucid3_player.htm]

Multimedia in the Lucid3 Player


# Multimedia in the Lucid3 Player

Many Lucid3 keys provide images and web pages for features, states and
entities, to help make an identification easier and more accurate and
to provide extra information on the items in the key. For example, web
pages and images attached to features and states may help you decide which
state of a feature to choose, or provide background hints and tips on
difficult features. Similarly, web pages and images attached to entities
may provide descriptions and extra information on the entities in the
key, both to add to the available information and to help you choose whether
an identification is correct.

By default, items in the Features and Entities panels that have images
attached will be preceded by the icon, while items that
have web pages attached will be preceded by the icon.   
  
  
Optionally, image thumbnails can be displayed in the Features and Entities
panels for items that have images attached. To display thumbnails check
the *Image Gallery* or *Image
Thumbnails* option located in the *Display*
submenu within the *Features* or
*Entities* menus.

With *Image Gallery* or *Image Thumbnails* checked features or
entities that have images attached will be preceded by a small thumbnail
of an image associated with that item. With *Image
Gallery* checked states are replaced with a tabular view of their
thumbnails, see *Feature Gallery View*
for more information.

To view a web page or image attached to an item click on its icon or
thumbnail.
